# Supplementary material for: Biological Response of Irisin Induced by Different Types of Exercise in Obese Subjects: A Non-Inferiority Controlled Randomized Study
Source: Biology (Basel). 2022 Mar 2;11(3):392. doi: 10.3390/biology11030392 (PMC8945525; doi:10.3390/biology11030392)
Supplement: Supplementary file 1 [file biology-11-00392-s001.zip › biology-1577696-supplementary.pdf]

Table S1. Variations of training measures/cardiorespiratory fitness, body composition and metabolic parameters before and after the 12-week training intervention program

Variable	Intervention Effect		
	BDC *Mean ± SD*	*PTDC* *Mean ± SD*	*Paired Samples Test* *P value*
**VO2 peak (ml)**	2952.5±736.1	3268.6±776.9	**.000**
**VO2 peak/weight (ml/kg)**	28.0±6.0	32.9±6.7	**.000**
**HR (bpm)**	72.7±10.7	62.0±9.8	**.000**
**SBP (mmHg)**	126.9±14.2	126.5±12.4	.818
**DBP mmHg)**	74.2±10.4	68.6±10.1	**.003**
**DAI (kcal/die)**	2058.9±652.1	1657.4±529.8	**.000**
**Weight (kg)**	105.3±14.4	99.4±14.4	**.000**
**BMI (kg/mq)**	35.6±4.4	33.6±4.5	**.000**
**Waist (cm)**	113.3±11.6	109.3±12.4	**.000**
**FM (kg)**	38.0±9.5	32.7±9.4	**.000**
**FM (%)**	36.5±8.3	33.2±8.8	**.000**
**FFM (kg)**	67.3±13.7	66.6±13.8	.240
**FFM (%)**	63.5±8.3	66.7±8.8	**.000**
**Glucose (mg/dl)**	98.7±9.3	96.9±8.8	.145
**Insulin (µU/ml)**	10.2±5.2	9.6±10.0	.651
**HOMA index**	2.5±1.4	2.4±2.8	.715
**IGF1 (ng/ml)**	207.2±76.3	208.9±74.6	.788
**Cortisol (ng/ml)**	96.5±34.2	85.6±25.9	.072
**hsCRP (mg/dl)**	.3±.4	.4±.5	.444
**Total-C (mg/dl)**	220.0±37.2	202.0±37.0	**.001**
**LDL-C (mg/dl)**	145.3±30.1	129.7±33.7	**.001**
**HDL-C (mg/dl)**	50.0±13.0	48.2±10.9	.163
**Triglycerides (mg/dl)**	123.6±71.4	120.9±72.6	.811
**Irisin (µg/ml)**	6.5±1.3	5.6±1.1	**.000**
**FNDC5 gene expression (RQ)**	1.3±.4	1.3±.6	.596

VO2, Volume of Oxygen; HR, Heart rate; SBP, Systolic Blood Pressure; DBP, Diastolic Blood Pressure; DCI, Daily Calorie Intake; BMI, Body Mass Index; Waist, Waist circumference; FM, Fat Mass; FFM, Free Fat Mass; HOMA IR index, HOmeostasis Model Assessment Insulin Resistance; IGF1, Insulin-like Growth Factor 1; hsCRP, high-sensitivity C-Reactive Protein; Total-C, Total Cholesterol; LDL-C, LDL Cholesterol calculated using Friedewald's Equation; HDL-C, HDL Cholesterol.
BDC, Baseline Data Collection; PTDC, Post Training Data Collection.
Data analysed with Paired Samples Test and expressed as means±SD.

Table S2. Bivariate Correlation Analyses between Irisin Plasma Levels or FNDC5 Gene Expression and Clinical Characteristics of Participants before and after the 12-week Training Program

		**Irisin (μg/ml)**						**FNDC5 gene expression (RQ)**					
		BDC		***PTDC***		***Change PTDC vs BDC***		***BDC***		***PTDC***		***Change PTDC vs BDC***	
		Rho	*P value*	*Rho*	*P value*	*Rho*	*P value*	*Rho*	*P value*	*Rho*	*P value*	*Rho*	*P value*
**Age (years)**		.065	.723	-.086	.644	-.157	.399	-.300	.114	-.534	.003	-.244	.211
**Education (years)**		.068	.710	.052	.780	.020	.914	-.134	.487	.246	.208	.199	.311
**Max. weight in adulthood (kg)**		-.257	.216	-.440	**.031**	-.201	.346	.229	.294	-.002	.992	-.119	.597
**Metabolic Syndrome score (n)**		-.059	.749	-.181	.331	.060	.749	.019	.924	.097	.625	.074	.709
		median [IQ range]		*median [IQ range]*		*median [IQ range]*		*median [IQ range]*		*median [IQ range]*		*median [IQ range]*	
**Sex**	***male***	5.6	**.005**	5.0	**.001**	-.5	.892	1.4	.988	1.1	.104	-.1	.164
	female	6.8		6.3		-.9		1.3		1.4		.1	
**Overweight/obesity in childhood**	***no***	6.19	.677	5.22	.482	-.51	.726	1.21	.392	1.28	.815	.17	.482
	yes	6.43		6.01		-.79		1.41		1.21		-.13	
**Overweight/obesity at puberty/menarche**	***no***	5.58	.158	4.79	.251	-.43	.581	1.15	.319	1.24	.704	.16	.649
	yes	6.47		5.85		-.82		1.41		1.22		-.07	
**Previous attempts to weight loss**	***no***	5.60	.238	5.44	**.877**	-.46	.725	2.82	**.016**	1.88	.455	-.18	.818
	yes	6.29		5.67		-.65		1.31		1.22		.06	
**Elevated Glucose criteria**	***no***	6.39	.455	5.88	.247	-.99	.520	1.33	.813	1.19	.910	-.12	.667
	yes	6.19		5.47		-.50		1.30		1.22		.10	
**Insulin Resistance according to HOMA IR model**	***<2.0***	6.04	.823	5.22	.653	-1.19	.200	1.29	.101	1.17	.599	.00	.956
	≥2.0	6.21		5.89		-.36		1.54		1.36		.07	
**Hypertension criteria**	***no***	6.58	.536	5.66	.830	-.50	.468	1.32	.897	1.15	.274	.06	.618
	yes	5.89		5.51		-.65		1.37		1.32		.11	
**Lipid criteria**	***no***	6.15	.716	5.72	.296	-.65	.751	1.32	.799	1.21	.745	.01	.672
	yes	6.37		4.93		-.51		1.44		1.17		.07	
**Metabolic Syndrome**	***<3.0***	6.15	**.863**	5.75	.164	-.50	.952	1.37	.512	1.17	.654	-.12	.598
	≥3.0	6.20		4.91		-.68		1.21		1.28		.10	

Metabolic Syndrome, revised NCEP ATP III criteria for Metabolic Syndrome*; Elevated Glucose criteria, revised NCEP/ATP III criteria for elevated Glucose (Fasting Glucose ≥100 mg/dl and/or on drug treatment for elevated glucose); Hypertension criteria, revised NCEP/ATP III criteria for hypertension (history of hypertension and/or drug treatment for elevated blood pressure and/or blood pressure ≥130/85 mm Hg); Lipid criteria, revised NCEP/ATP III criteria for Lipid (TG ≥150 mg/dL and/or HDL-C <40 mg/dL in male or <50 mg/dL in female or On drug treatment for elevated triglycerides); BDC, Baseline Data Collection; PTDC, Post Training Data Collection. Data are expressed as Spearman's Rho or median [IQ, interquartile range] unless otherwise stated.

*Grundy SM, Cleeman JI, Daniels SR, Donato KA, Eckel RH, Franklin BA, Gordon DJ, Krauss RM, Savage PJ, Smith SC Jr. et al. Diagnosis and management of the metabolic syndrome: an American Heart Association/National Heart. Lung. and Blood Institute Scientific Statement. Circulation. 2005;112(17):2735–52.

Table S3. Bivariate Correlation Analyses between Irisin Plasma Levels or FNDC5 Gene Expression and Physical capacities/Training measures

		**Irisin (µg/ml)**						**FNDC5 gene expression (RQ)**					
		BDC		***PTDC***		***Change PTDC vs BDC***		***BDC***		***PTDC***		***Change PTDC vs BDC***	
		Rho	*P value*	*Rho*	*P value*	*Rho*	*P value*	*Rho*	*P value*	*Rho*	*P value*	*Rho*	*P value*
**Number session (n)**				.413	**.021**	.298	.104			.210	.283	.043	.828
**Time for session (min)**				-.228	.217	.030	.871			.027	.891	.061	.759
**EEE/session (kcal/session)**				-.422	**.018**	.091	.626			-.198	.313	-.150	.447
**EEE/minute (kcal/min)**				-.138	.459	.035	.850			-.204	.297	-.183	.352
**EEE/weight/session (kcal/kg/session)**				.141	.450	.098	.600			.082	.678	.077	.698
**VO_2 peak (ml)**	***BDC***	-.374	**.035**	-.422	**.018**	.088	.637	-.062	.751	-.196	.316	-.193	.326
	PTDC			-.372	**.039**	.139	.455			-.214	.274	-.150	.447
	Change PTDC vs BDC			-.036	.846	.078	.676			-.115	.558	.038	.849
**VO_2 peak/weight (ml/kg)**	***BDC***	-.243	.180	-.286	.119	.082	.660	-.063	.747	-.186	.344	-.203	.300
	PTDC			-.240	.194	.104	.578			-.253	.194	-.187	.342
	Change PTDC vs BDC			-.027	.884	.062	.742			-.247	.205	-.135	.493
**HR (bpm)**	***BDC***	-.042	.821	.258	.162	.363	.045	-.024	.903	.301	.120	.301	.120
	PTDC			.305	.095	.267	.147			.030	.881	.062	.754
	Change PTDC vs BDC			.014	.941	-.180	.333			-.395	**.037**	-.301	.120
**SBP (mmHg)**	***BDC***	-.177	.331	-.109	.559	.059	.754	.141	.467	.090	.649	-.073	.710
	PTDC			.207	.265	.159	.392			.208	.289	.133	.499
	Change PTDC vs BDC			.454	**.010**	.055	.767			.136	.489	.188	.337
**DBP (mmHg)**	***BDC***	-.141	.440	-.032	.863	.158	.396	-.077	.690	-.076	.702	-.062	.752
	PTDC			-.074	.691	-.107	.566			.009	.965	.001	.994
	Change PTDC vs BDC			-.019	.921	-.228	.218			-.027	.891	-.052	.795
**DCI (kcal/die)**	***BDC***	-.171	.349	-.152	.416	.096	.608	.168	.384	.070	.725	-.157	.426
	PTDC			-.221	.231	.134	.473			.068	.729	.011	.954
	Change PTDC vs BDC			-.136	.465	.080	.669			.037	.853	.342	.075

EEE, Energy Expenditure Exercise; VO2, Volume of Oxygen; HR, Heart rate; SBP, Systolic Blood Pressure; DBP, Diastolic Blood Pressure; DCI, Daily Calorie Intake. BDC, Baseline Data Collection; PTDC, Post Training Data Collection. Data are expressed as Spearman's Rho.

Table S4. Bivariate Correlation Analyses between Irisin Plasma Levels or FNDC5 Gene Expression and Anthropometric and Body Composition Measures

		**Irisin (µg/ml)**						**FNDC5 gene expression (RQ)**					
		BDC		***PTDC***		***Change PTDC vs BDC***		***BDC***		***PTDC***		***Change PTDC vs BDC***	
		Rho	*P value*	*Rho*	*P value*	*Rho*	*P value*	*Rho*	*P value*	*Rho*	*P value*	*Rho*	*P value*
**Weight (kg)**	***BDC***	-.356	**.045**	-.330	.070	.070	.709	.067	.730	-.018	.929	-.014	.944
	PTDC			-.296	.106	.030	.871			.090	.649	.093	.637
	Change PTDC vs BDC			.126	.501	-.038	.840			.249	.202	.210	.284
**BMI (kg/mq)**	***BDC***	-.019	.917	.271	.140	.299	.102	.156	.418	.183	.350	.094	.636
	PTDC			.247	.180	.229	.215			.228	.243	.124	.529
	Change PTDC vs BDC			-.018	.925	-.077	.680			.241	.216	.227	.246
**Waist (cm)**	***BDC***	-.252	.164	-.092	.623	.123	.511	.091	.638	-.259	.183	-.257	.187
	PTDC			-.025	.895	.224	.225			-.136	.490	-.230	.239
	Change PTDC vs BDC			.179	.336	.177	.342			.211	.282	.093	.640
**FM (kg)**	***BDC***	.118	.520	.210	.257	.060	.746	.267	.162	.463	**.013**	.221	.259
	PTDC			.255	.166	-.008	.966			.434	**.021**	.218	.265
	Change PTDC vs BDC			.057	.760	-.205	.267			.253	.194	.221	.259
**FM (%)**	***BDC***	.379	**.032**	.432	**.015**	-.064	.732	.168	.384	.506	**.006**	.359	.061
	PTDC			.419	**.019**	-.080	.668			.460	**.014**	.260	.182
	Change PTDC vs BDC			.092	.621	-.148	.427			.253	.194	.230	.239
**FFM (kg)**	***BDC***	-.454	**.009**	-.518	**.003**	.072	.700	-.092	.634	-.298	.124	-.204	.299
	PTDC			-.490	**.005**	.083	.658			-.236	.227	-.163	.407
	Change PTDC vs BDC			-.016	.930	-.015	.936			.264	.175	.175	.372
**FFM (%)**	***BDC***	-.376	**.034**	-.432	**.015**	.064	.732	-.174	.367	-.506	**.006**	-.359	.061
	PTDC			-.418	**.019**	.085	.649			-.428	**.023**	-.224	.252
	Change PTDC vs BDC			-.063	.737	.157	.399			-.175	.373	-.165	.402

BMI, Body Mass Index; Waist, Waist circumference; FM, Fat Mass; FFM, Free Fat Mass. BDC, Baseline Data Collection; PTDC, Post Training Data Collection. Data are expressed as Spearman's Rho.

Table S5. Bivariate Correlation Analyses between Irisin Plasma Levels or FNDC5 Gene Expression and Metabolic Measures

		Irisin (µg/ml)						FNDC5 gene expression (RQ)					
		BDC		*PTDC*		*Change PTDC vs BDC*		*BDC*		*PTDC*		*Change PTDC vs BDC*	
		Rho	*P value*	Rho	*P value*	Rho	*P value*	Rho	*P value*	Rho	*P value*	Rho	*P value*
**Glucose (mg/dl)**	***BDC***	-.057	.758	-.005	.980	.257	.163	-.194	.313	.085	.669	.172	.380
	PTDC			.023	.903	.107	.567			.105	.593	.217	.268
	Change PTDC vs BDC			.024	.899	-.200	.280			.028	.888	.003	.989
**Insulin (µU/ml)**	***BDC***	-.093	.612	.066	.725	.322	.077	.136	.483	.136	.489	.040	.840
	PTDC			-.095	.611	.174	.348			.094	.635	.021	.914
	Change PTDC vs BDC			-.123	.510	-.007	.968			-.080	.685	-.108	.584
**HOMA IR index**	***BDC***	-.127	.488	.029	.877	.357	**.049**	.106	.583	.210	.284	.132	.503
	PTDC			-.130	.484	.187	.313			.086	.664	.026	.894
	Change PTDC vs BDC			-.142	.445	-.056	.766			-.101	.610	-.131	.505
**IGF*1* (ng/ml)**	***BDC***	.068	.711	-.177	.340	-.231	.210	-.010	.960	.313	.105	.327	.089
	PTDC			-.197	.288	-.041	.826			.273	.161	.336	.080
	Change PTDC vs BDC			.243	.188	.415	**.020**			.159	.418	.122	.538
**Cortisol (ng/ml)**	***BDC***	-.057	.757	-.321	.078	-.135	.467	.084	.666	-.094	.636	-.139	.480
	PTDC			.125	.503	-.105	.575			-.031	.875	.171	.385
	Change PTDC vs BDC			.440	**.013**	.075	.687			.152	.440	.414	**.029**
***hs*CRP (mg/dl)**	***BDC***	.355	**.046**	.280	.127	-.116	.534	-.109	.572	.360	.060	.367	.054
	PTDC			.155	.406	-.174	.348			.269	.167	.169	.391
	Change PTDC vs BDC			-.260	.157	-.124	.507			-.069	.727	-.142	.472
**Total-C (mg/dl)**	***BDC***	.018	.921	-.053	.778	.163	.382	-.226	.239	-.163	.407	-.084	.670
	PTDC			.042	.821	.024	.897			.005	.980	.170	.388
	Change PTDC vs BDC			.076	.684	-.143	.442			.140	.477	.201	.304
**LDL-C (mg/dl)**	***BDC***	.063	.732	-.094	.615	.073	.696	-.245	.201	-.284	.144	-.171	.385
	PTDC			-.040	.829	-.049	.794			-.151	.445	.038	.847
	Change PTDC vs BDC			-.109	.560	-.221	.232			.117	.553	.282	.145
**HDL-C (mg/dl)**	***BDC***	.210	.249	.365	**.044**	.081	.663	.033	.863	.188	.337	.125	.527
	PTDC			.515	**.003**	.084	.653			.200	.307	.195	.319
	Change PTDC vs BDC			.193	.298	-.132	.478			.054	.784	-.022	.910
**Triglycerides (mg/dl)**	***BDC***	.007	.970	-.183	.324	-.051	.786	-.237	.215	.007	.971	.025	.901
	PTDC			-.202	.276	-.094	.614			-.106	.593	-.130	.511
	Change PTDC vs BDC			.109	.560	.013	.943			-.005	.980	.012	.952

HOMA IR index, HOmeostasis Model Assessment Insulin Resistance; IGF1, Insulin-like Growth Factor 1; hsCRP, high-sensitivity C-Reactive Protein; Total-C, Total Cholesterol; LDL-C, LDL Cholesterol calculated using Friedewald's Equation; HDL-C, HDL Cholesterol. BDC, Baseline Data Collection; PTDC, Post Training Data Collection. Data are expressed as Spearman's Rho.

Table S6. Changes in anthropometrics, body composition, training and metabolic measures stratified by median level of irisin change after 12-week of training program among obese healthy participants.

Measures	Group	Intervention		Intervention Effect			Intervention x Group effect		
	above/below median change Irisin	*BDC Mean±SD*	*PTDC Mean±SD*	*Change within group MD (95% CI)*	*P value*	*Partial η2*	*Change between group MD (95% CI)*	*P value*	*Partial η2*
**VO2 peak (ml)**	***below median***	2852.4±753.0	3133.5±756.8	300.5 (211.5 – 389.4)	.0001	.622	281.1 (173.3 – 388.9)	.659	.007
	above median	3059.2±754.1	3379.0±819.0				319.8 (165.6 – 474.1)		
**VO2 peak/weight (ml/kg)**	***below median***	27.7±6.9	32.1±7.4	4.8 (3.6 – 5.9)	.0001	.713	4.4 (3.0 – 5.8)	.570	.011
	above median	28.6±5.2	33.6±6.2				5.1 (3.1 – 7.1)		
**HR (bpm)**	***below median***	68.5±9.7	59.0±8.2	-10.8 (-13.9 – -7.6)	.0001	.626	-9.5 (-14.7 – -4.3)	.414	.023
	above median	77.3±10.1	65.2±10.7				-12.1 (-16.0 – -8.2)		
**DBP mmHg)**	***below median***	73.4±10.5	70.3±9.8	-5.7 (-9.2 – -2.2)	.002	.282	-3.2 (-8.3 – 2.0)	.142	.073
	above median	75.1±10.6	66.8±10.4				-8.3 (-13.4 – -3.1)		
**DAI (kcal/die)**	***below median***	2022.7±657.1	1592.4±578.4	-411.0 (-625.2 – -196.7)	.0001	.347	-430.3 (-712.6 – -148.0)	.855	.001
	above median	2143.3±660.5	1751.6±488.5				-391.6 (-743.3 – -39.9)		
**Weight (kg)**	***below median***	103.3±13.3	98.1±13.0	-5.9 (-7.8 – -4.0)	.0001	.583	-5.3 (-8.0 – -2.5)	.494	.016
	above median	106.8±16.1	100.3±16.5				-6.5 (-9.3 – -3.7)		
**BMI (kg/mq)**	***below median***	34.7±4.4	33.0±4.4	-2.0 (-2.7 – -1.4)	.0001	.583	-1.8 (-2.7 – -.8)	.460	.019
	above median	36.5±4.4	34.2±4.6				-4.1 (-17.6 – 25.8)		
**Waist (cm**	***below median***	111.2±9.0	106.4±8.2	-5.1 (-13.7 – 3.5)	.0001	.425	-4.8 (-7.0 – -2.6)	.370	.028
	above median	115.6±13.9	112.3±15.4				-3.2 (-6.3 – -.2)		
**FM (kg)**	***below median***	37.8±11.0	33.3±10.3	-5.5 (-7.3 – -3.7)	.0001	.565	-4.5 (-7.0 – -2.0)	.265	.043
	above median	38.6±8.4	32.1±9.1				-6.5 (-9.4 – -3.6)		
**FM (%)**	***below median***	36.6±9.4	34.0±9.3	-3.4 (-4.7 – -2.0)	.0001	.475	-2.6 (-4.0 – -1.1)	.235	.048
	above median	36.5±7.3	32.3±8.5				-4.2 (-6.6 – -1.7)		
**FFM (kg)**	***below median***	65.7±13.6	64.7±13.3	-.5 (-1.7– .6)	.360	.029	-1.0 (-1.9 – -.1)	.398	.025
	above median	68.2±14.3	68.2±14.9				.0 (-2.3 – 2.2)		
**FFM (%)**	***below median***	63.4±9.5	65.8±9.2	3.3 (1.9 – 4.7)	.0001	.448	2.4 (.9 – 3.9)	.197	.057
	above median	63.5±7.3	67.7±8.5				4.2 (1.7 – 6.7)		
**Total-C (mg/dl)**	***below median***	220.0±27.6	204.6±30.6	-18.1 (-28.3 – -7.9)	.001	.313	-15.4 (-31.2 – .4)	.592	.010
	above median	220.0±46.4	199.3±43.7				-20.8 (-34.9 – -6.7)		
**LDL-C (mg/dl)**	***below median***	146.9±25.4	132.8±28.4	-15.6 (-24.3 – -6.9)	.001	.316	-14.1 (-27.7 – -.5)	.727	.004
	above median	143.5±35.2	126.4±39.4				-17.1 (-29.1 – -5.1)		

VO2, Volume of Oxygen; HR, Heart rate; DBP, Diastolic Blood Pressure; DCI, Daily Calorie Intake; BMI, Body Mass Index; Waist, Waist circumference; FM, Fat Mass; FFM, Free Fat Mass; Total-C, Total Cholesterol; LDL-C, LDL Cholesterol calculated using Friedewald's Equation. BDC, Baseline Data Collection; PTDC, Post Training Data Collection. Analysis performed with General Linear Model (GLM) Repeated Measures, Within-Subjects and Between-Subjects test. Mean ± standard deviation (SD); median deviation (MD) and 95% confidence intervals (95% CI).
